# Local structure, nucleation sites and crystallization behavior and their effects on magnetic properties of Fe_81_Si_*x*_B_10_P_8−*x*_Cu_1_ (*x* = 0~8)

**DOI:** 10.1038/s41598-018-19665-8

**Published:** 2018-01-19

**Authors:** C. C. Cao, Y. G. Wang, L. Zhu, Y. Meng, X. B. Zhai, Y. D. Dai, J. K. Chen, F. M. Pan

**Affiliations:** 1 College of Materials Science and Technology, Nanjing University of Aeronautics and Astronautics, Nanjing, 211106 P. R. China; 2 College of Science, Nanjing University of Aeronautics and Astronautics, Nanjing, 211106 P. R. China

## Abstract

In this work, an attempt has been made to reveal critical factors dominating the crystallization and soft magnetic properties of Fe_81_Si_*x*_B_10_P_8−*x*_Cu_1_ (*x* = 0, 2, 4, 6 and 8) alloys. Both melt spun and annealed alloys are characterized by differential scanning calorimetry, X-ray diffractometry, Mössbauer spectroscopy, transmission electron microscopy, positron annihilation lifetime spectroscopy and magnetometry. The changes in magnetic interaction between Fe atoms and chemical homogeneity can well explain the variation of magnetic properties of Fe_81_Si_*x*_B_10_P_8−*x*_Cu_1_ amorphous alloys. The density of nucleation sites in the amorphous precursors decreases in the substitution of P by Si. Meanwhile, the precipitated nanograins gradually coarsen, but the inhibiting effect of P on grain growth diminishes causing the increase of the crystallinity. Moreover, various site occupancies of Si are observed in the nanocrystallites and the Si occupancy in *bcc* Fe decreases the average magnetic moment of nanograins. Without sacrificing amorphous forming ability, we can obtain FeSiBPCu nanocrystalline alloy with excellent soft magnetic properties by optimizing the content of Si and P in the amorphous precursors.

## Introduction

Fe-based nanocrystalline soft magnetic alloys with the amorphous/crystalline composite structures have become a hot topic in both research and application. These alloys usually possess outstanding magnetic properties involving high saturation magnetic flux density (*B*_S_) and low coercivity (*H*_C_)^1–4^. Current state of the art nanocrystalline alloys mainly include FeSiBNbCu^5^, FeSiBPCu^6^, FeMBCu (M = Zr, Nb, Hf)^7^ and their derivatives which contain a handful of early transition metal elements to promote their crystallization^8^. It is noteworthy that soft magnetic properties of these alloys are usually variable even if the atomic percent of magnetic Fe atoms keeps the same. For example, Fe_82.65_Cu_1.35_Si_2_B_14_ nanocrystalline alloy shows an *H*_C_ of 6.5 A/m and a *B*_S_ of 1.84 T, while the *H*_C_ and *B*_S_ of Fe_82.65_Cu_1.35_Si_5_B_11_ are 60 A/m and 1.81 T, respectively^9^. Magnetic properties of nanocrystalline alloys are closely associated with the amount, size and chemical composition of nanograins embedded in the amorphous matrix.

Every kind of metalloid and transition metal element plays an essential role on the nanocrystallization and only with appropriate content can the magnetic properties be improved. It has been proven that moderate addition of Cu can provide nucleation sites for α-Fe and thus refines the primary nanocrystallites due to the formation of Cu clusters^10,11^. Moreover, the differences in the kind and content of solute elements usually result in various structures and chemical compositions of nanograins. For instance, α-Fe phase with *bcc* structure precipitates out in the Fe_83.3_Si_4_B_8_P_4_Cu_0.7_ alloy^12^, but Fe_3_Si phase with DO_3_ structure precipitates out in the Fe_73.5_Si_13.5_B_9_Nb_3_Cu_1_ alloy^13^. The composition of precipitated nanograins varies with the content of Si even in the same system, such as in FeSiBCu^14^ and FeSiBNbCu alloys^15^, respectively. In addition, the co-addition of Cu and P can further refine the nanograins, which results from the interaction between solute atoms forming CuP clusters^16^. The crystallization process tightly contacts with the microstructure of amorphous precursors including short range order, free volume, chemical homogeneity and particularly nucleation sites. Thus comprehensively understanding the transformation of amorphous precursors during the crystallization appears to be significantly important for advanced materials design.

For those nanocrystalline alloys with heterogeneous nucleation, various methods have been proposed to increase nucleation sites for the refinement of nanograins, such as the addition of Cu into the FeZrNbB alloy^17^. When the amorphous precursors contain insoluble atoms or atom pairs, such as Cu, Nb and Cu-P, they tend to precipitate out and agglomerate to form insoluble clusters at the early stage of annealing process which can serve as nucleation sites for nanograins. However, little is known about the real content of nucleation sites during the crystallization which is usually reflected by the size of nanograins. It is probable to be inaccurate in some cases because some solute atoms such as P can also restrain the growth of nanograins^18^. The difficulty lies in the facts that nucleation sites are too small to be characterized and that one can hardly discern which clusters can effectively serve as nucleation sites. But from another point of view, crystallites precipitate out on nucleation sites at the very beginning of crystallization with the formation of nanovoids at grain boundaries^19^. Positron annihilation lifetime spectroscopy (PALS) is an efficient method to provide precise information about characteristic positron annihilation sites in nanocrystalline alloys, such as interstitial holes, vacancies and grain boundaries^20,21^. This technology can be used to characterize the formation and growth of crystallites, which can effectively reflect the density of nucleation sites during the crystallization. Meanwhile, Mössbauer spectroscopy can precisely identify the local structure around atomic nucleus for its high energy resolution, with which the information about short-range order in the amorphous matrix and chemical compositions of the nanograins can be obtained^22^.

In this work, we analyze the local structure differences in both melt spun and annealed Fe_81_Si_*x*_B_10_P_8−*x*_Cu_1_ (*x* = 0, 2, 4, 6 and 8) alloys to reveal the critical factors dominating the crystallization. Specifically, we focus on the microstructure of amorphous precursors, nucleation sites at the beginning of crystallization and crystal phases after the nanocrystallization. Furthermore, we are aiming at understanding the effects of local structure, nucleation sites and crystallization behavior of the amorphous precursors on magnetic properties of nanocrystalline alloys.

## Results and Discussion

### Thermal properties

**Figure 1:**
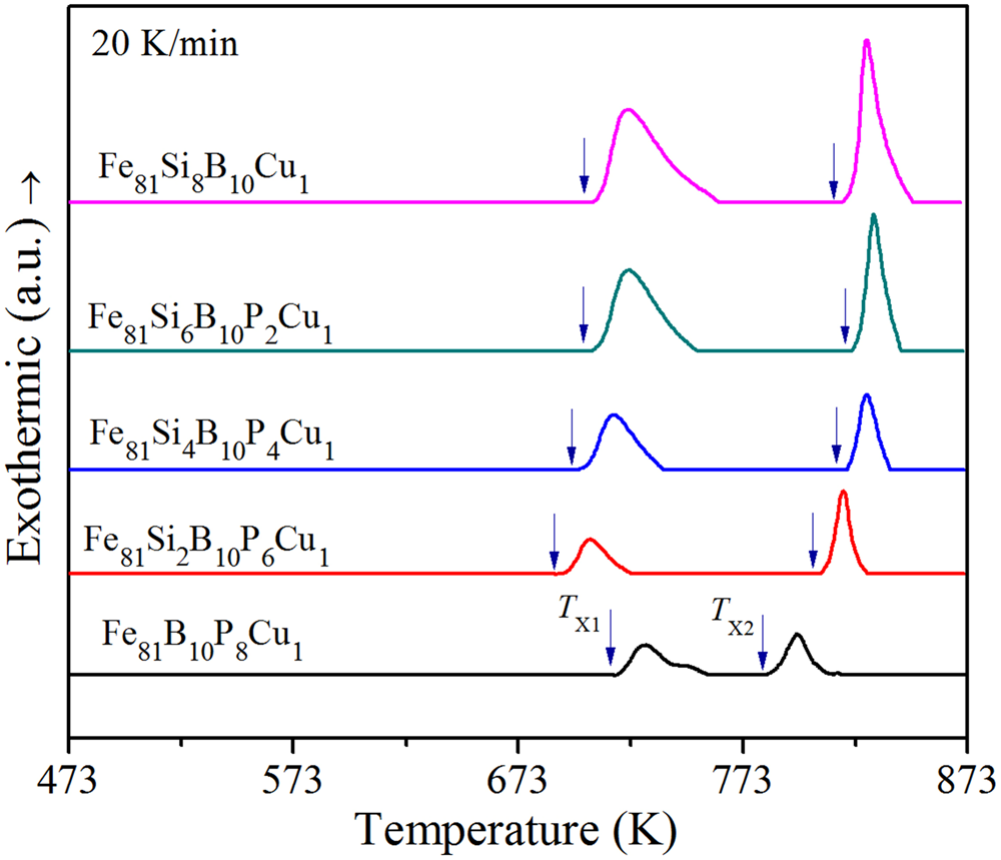
DSC curves of melt spun Fe_81_Si_*x*_B_10_P_8−*x*_Cu_1_ (*x* = 0~8) ribbons.

As shown in Fig. 1, differential scanning calorimetry (DSC) curves are collected to investigate the thermal dynamic characteristics of Fe_81_Si_*x*_B_10_P_8−*x*_Cu_1_ (*x* = 0, 2, 4, 6 and 8) alloys. All these alloys have two conspicuous and separated exothermic peaks marked as *T*_X1_ and *T*_X2_, which indicates that the crystallization of Fe_81_Si_*x*_B_10_P_8−*x*_Cu_1_ alloys occurs in two stages. For Fe_81_Si_*x*_B_10_P_8−*x*_Cu_1_ alloys with *x* = 0, 2, 4, 6 and 8, *T*_X1_ is 713, 688, 697, 701 and 704 K, respectively. It is worth noting that 2at. % of Si significantly advances the primary crystallization but postpones the secondary crystallization, and then both *T*_X1_ and *T*_X2_ increase gradually with Si further substituting for P. The temperature interval (Δ*T*_X_ = *T*_X2_ − *T*_X1_) between the two peaks shows a maximum of about 117 K for Fe_81_Si_4_B_10_P_4_Cu_1_ alloy. The smaller Δ*T*_X_ means the more strict annealing treatment during the crystallization process, especially for Fe_81_B_10_P_8_Cu_1_ alloy in which the secondary crystallization practically tends to occur. Thus, appropriate Si superseding P in Fe_81_Si_*x*_B_10_P_8−*x*_Cu_1_ alloys provides an advantage for the nanocrystallization.

### Local structure in amorphous precursors and crystal phases

**Figure 2:**
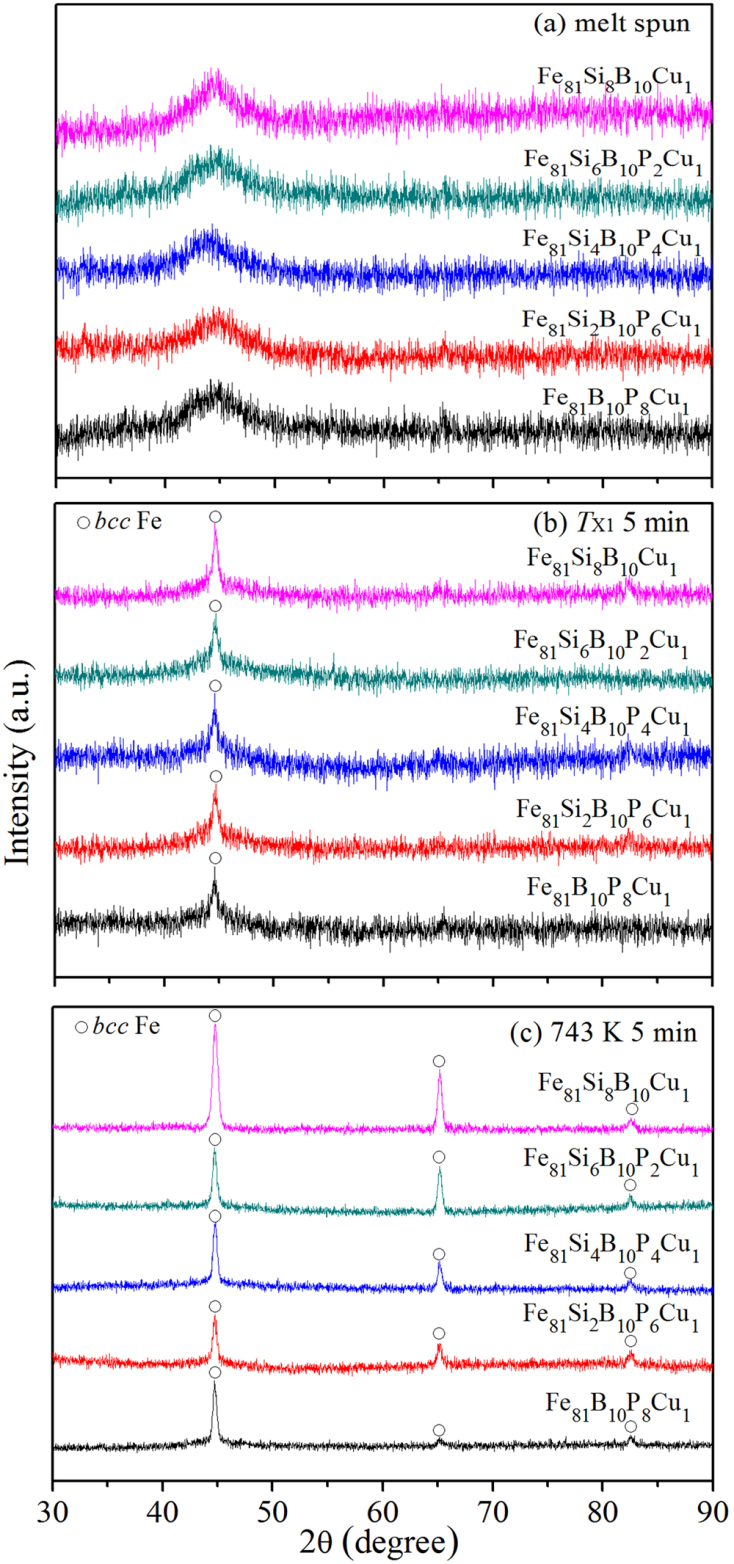
XRD patterns of Fe_81_Si_*x*_B_10_P_8−*x*_Cu_1_ (*x* = 0~8) melt spun ribbons (**a**) and those annealed at *T*_X1_ (**b**) and 743 K (**c**) for 5 min.

Figure 2 shows the X-ray diffractometry (XRD) patterns for the melt spun and annealed Fe_81_Si_*x*_B_10_P_8−*x*_Cu_1_ (*x* = 0, 2, 4, 6 and 8) alloys. Each diffraction pattern of the melt spun ribbons in Fig. 2(a) exhibiting a typical broad halo without any appreciable crystalline peak indicates the formation of amorphous structure. According to the results of DSC, all Fe_81_Si_*x*_B_10_P_8−*x*_ alloy ribbons are annealed at their individual *T*_X1_ for 5 min. As shown in Fig. 2(b), a weak crystalline peak located at 2θ = 45° corresponding to *bcc* Fe suggests the beginning of primary crystallization. Figure 2(c) presents the diffraction patterns of the Fe_81_Si_*x*_B_10_P_8−*x*_Cu_1_ nanocrystalline alloys obtained by annealing their amorphous precursors at 743 K for 5 minutes. In each pattern, there appear three distinct diffraction peaks corresponding to *bcc* Fe phase. The intensity of these peaks is enhanced as Si content x increases, which implies that the crystallinity of Fe_81_Si_*x*_B_10_P_8−*x*_Cu_1_ alloys increases with P gradually replaced by Si. Meanwhile, according to the Scherrer equation, the average mean grain size of the α-Fe phase is estimated to increase.

**Figure 3:**
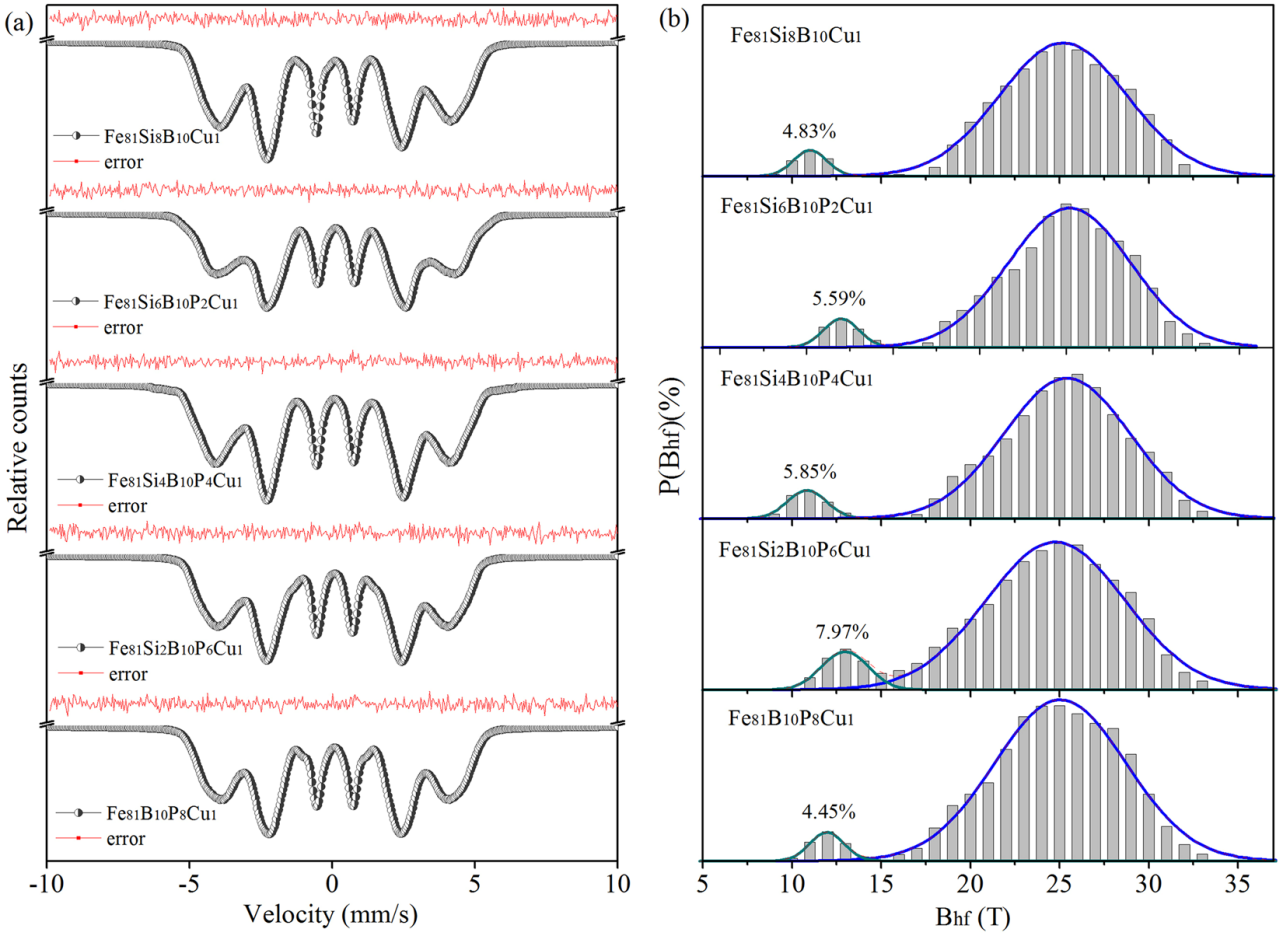
Mössbauer spectra (**a**) and corresponding hyperfine field distributions (**b**) of Fe_81_Si_*x*_B_10_P_8−*x*_Cu_1_ (*x* = 0~8) melt spun ribbons.

A further study with Mössbauer spectroscopy provides more detailed information about the microstructure of as-quenched and nanocrystalline Fe_81_Si_*x*_B_10_P_8−*x*_Cu_1_ (*x* = 0, 2, 4, 6 and 8) alloys. As shown in Fig. 3(a), the wide and asymmetrical sextets demonstrate the amorphous nature of all the melt spun alloys^23^. The hyperfine parameters of fitted Mössbauer spectra are given in Table 1. Isomer shift (*IS*) refers to the observed shift of the resonance spectrum, quadrupole splitting (*QS*) results from the asymmetrical distribution of charge around atomic nucleus, and isomer shift change (*DTI*) is a coupling parameter that ensures the observed asymmetry of the experimental spectrum. The average magnetic hyperfine field (*B*_hf,a_) is a parameter to describe the average hyperfine interaction and proportional to the spin-exchange interaction between magnetic atoms in each sample. It is noted that both Fe_81_B_10_P_8_Cu_1_ and Fe_81_Si_4_B_10_P_4_Cu_1_ show relatively high *B*_hf,a_ compared with the other amorphous alloys. As reported early, the strengthened magnetic interaction can be ascribed to an increased degree of order in the topological structure^24^.

**Table 1:** Hyperfine parameters of melt spun Fe_81_Si_*x*_B_10_P_8−*x*_Cu_1_ (*x* = 0~8) ribbons: average magnetic hyperfine field (*B*_hf,a_), change of isomer shift (*DTI*), Isomer shift relative to α-Fe (*IS*), quadrupole splitting (*QS*).

Samples	*IS* (mm/s)	*DTI* (mm/s)	*QS* (mm/s)	*B*_hf,a_ (T)
Fe_81_B_10_P_8_Cu_1_	0.104	0.0001	−0.0040	24.7
Fe_81_Si_2_B_10_P_6_Cu_1_	0.043	0.0010	−0.0641	24.2
Fe_81_Si_4_B_10_P_4_Cu_1_	0.096	0.0019	0.0032	24.5
Fe_81_S_6_B_10_P_2_Cu_1_	0.027	0.0021	−0.0661	23.9
Fe_81_Si_8_B_10_Cu_1_	−0.079	0.0073	0.0361	23.7

Hyperfine field distributions obtained from the Mössbauer spectra of melt spun Fe_81_Si_*x*_B_10_P_8−*x*_Cu_1_ alloys are depicted in Fig. 3(b). Magnetic hyperfine field distributions are usually decomposed into several components adopting Gaussian distributions to investigate the chemical short range orders in the structural relaxation or the crystal phases in the crystallization^25–27^. However, it is less accurate for melt spun metallic glasses where excess free volume and residual stress distort the coordination environment of Fe atoms. Nevertheless, all the hyperfine field distributions distinctly separate into two regions in Fig. 3(b) which suggests the coexistence of two distinguished zones in the amorphous structure of melt spun ribbons, namely the low field region (8 T to 15T) with weakened magnetic interactions and the high field region (15 T to 35 T) with enhanced magnetic interactions^28^. The low field region indicates Fe-deficient zones with plentiful B atoms and a little Cu atoms occupying in the neighbor shell of Fe atoms, while the high field region implies Fe enriched ones^29,30^. Therefore, the relative area of the low field region in Fig. 3(b) can be utilized to evaluate the chemical homogeneity of the amorphous structure. The maximum area ratio of the low field region in Fe_81_Si_2_B_10_P_6_Cu_1_ alloy manifests a high degree of inhomogeneity in the amorphous structure which will promote the crystallization in the annealing, while the minimum value of that in Fe_81_B_10_P_8_Cu_1_ alloy leads to a high degree of homogeneity and a high *T*_x1_, which is consistent with the results from DSC.

**Figure 4:**
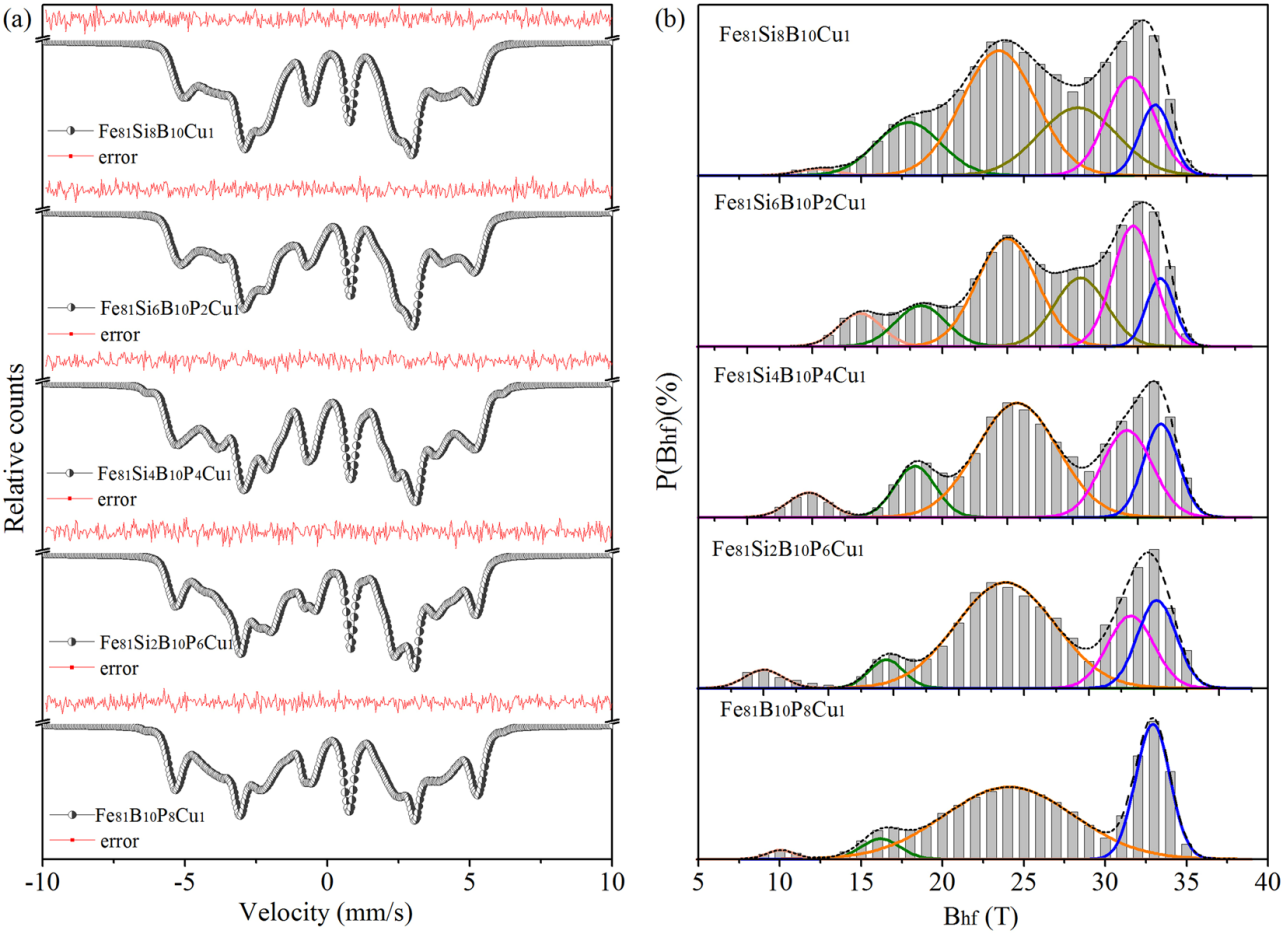
Mössbauer spectra (**a**) and corresponding hyperfine field distributions (**b**) of Fe_81_Si_*x*_B_10_P_8−*x*_Cu_1_ (*x* = 0~8) nanocrystalline ribbons.

Figure 4 displays Mössbauer spectra and the corresponding hyperfine field distributions of Fe_81_Si_*x*_B_10_P_8−*x*_Cu_1_ nanocrystalline alloys obtained by annealing their amorphous precursors at 743 K for 5 min. The split sextets superimposed upon the broadened spectra in Fig. 4(a) and the emergence of split peaks at about 33 T corresponding to *bcc* Fe in Fig. 4(b) characterize the microstructure with nanograins embedded in the residual amorphous matrix^31^. As reported previously^32^, crystal phases in FeSi alloys (<10 at.% Si) prefer to form *bcc* Fe structure during the crystallization where the central Fe atom has 6~8 nearest-neighbor Fe atoms with Si atoms occupying at the residual sites. The *bcc* Fe structure with all the eight nearest sites occupied by Fe atoms is defined as an A8 configuration. Similarly, A7 and A6 configuration mean the *bcc* Fe structure with seven and six nearest-neighbor Fe atoms, respectively. Therefore, to further explore the specific site occupancy in the nanocrystallites of Fe_81_Si_*x*_B_10_P_8−*x*_Cu_1_ alloys, the hyperfine field distributions are precisely decomposed into several components by Gaussian distributions. The gray histograms in Fig. 4(b) represent the actual hyperfine field distributions, while the dotted black lines represent the fitting results obtained from the corresponding colored components. The excellent match between them confirms that the Gaussian fitting results can provide accurate structural information of the FeSiBPCu nanocrystalline alloys. Peaks at hyperfine fields of about 33 T, 31.5 T and 28.5 T can be attributed to A8, A7 and A6 configurations, respectively, as suggested by previous studies^14,33^. The other peaks in Fig. 4(b) can be assigned to Fe atoms with various coordination surroundings in the residual amorphous matrix. It is noteworthy that only nanograins with A8 configuration precipitate out in the Fe_81_B_10_P_8_Cu_1_ alloy because of the lack of Si, but for the rest alloys containing Si, both A8 and A7 configurations appear in the crystal phases resulting in the broadening or splitting of peaks. When *x* reaches 6, all the three configurations coexist in the *bcc* Fe crystal phases. The area ratio of each sub-peak and the sum of those corresponding to *bcc* Fe are shown in Table 2. With P gradually replaced by Si in the Fe_81_Si_*x*_B_10_P_8−*x*_Cu_1_ alloys, Si atoms tend to occupy more nearest sites of Fe atoms in *bcc* Fe structure, which results in the nanocrystallites diversified and the crystallinity of alloy ribbons increasing. It can be attributed to two aspects. On one hand, Si is preferentially spread into *bcc* nanocrystallites compared with amorphous matrix during the primary crystallization^14,32^. On the other hand, the decrease of P in the amorphous matrix weakens its inhibiting effect on grain growth^34^.

**Table 2:** Area ratios of sub-peaks corresponding to A8, A7, A6 and *bcc* Fe in hyperfine field distributions of nanocrystalline Fe_81_Si_*x*_B_10_P_8−*x*_Cu_1_ (*x* = 0~ 8) ribbons.

Samples	A8 (%)	A7 (%)	A6 (%)	*bcc* Fe (%)
Fe_81_B_10_P_8_Cu_1_	31.79			31.79
Fe_81_Si_2_B_10_P_6_Cu_1_	18.02	17.71		35.73
Fe_81_Si_4_B_10_P_4_Cu_1_	16.27	23.62		39.89
Fe_81_S_6_B_10_P_2_Cu_1_	9.30	24.43	17.53	51.26
Fe_81_Si_8_B_10_Cu_1_	7.78	21.92	23.48	53.18

**Figure 5:**
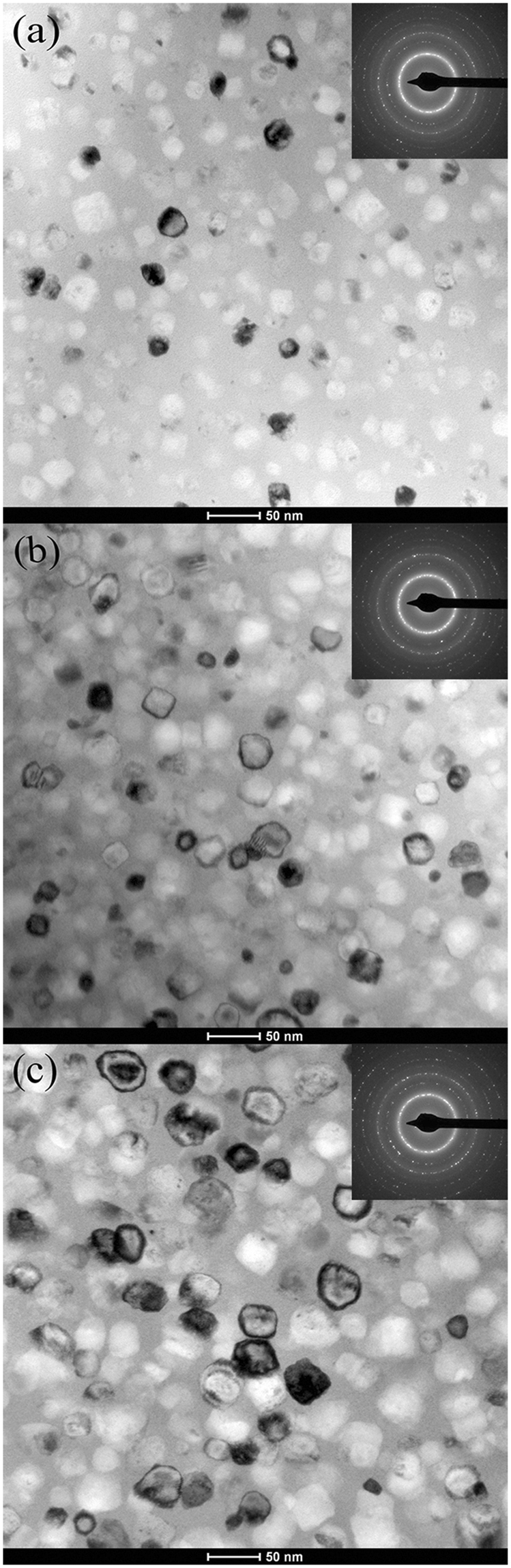
TEM bright field images and corresponding SAED patterns of (**a**) Fe_81_B_10_P_8_Cu_1_, (**b**) Fe_81_Si_4_B_10_P_4_Cu_1_ and (**c**) Fe_81_Si_8_B_10_Cu_1_ ribbons annealed at 743 K for 5 min.

As shown in Fig. 5, the bright field images of transmission electron microscopy (TEM) and the corresponding selected area electron diffraction (SAED) patterns prove the amorphous/nanocrystalline composite structures of the 743 K annealed Fe_81_B_10_P_8_Cu_1_, Fe_81_Si_4_B_10_P_4_Cu_1_ and Fe_81_Si_8_B_10_Cu_1_ ribbons, and the average grain sizes are 20 nm, 25 nm and 36 nm, respectively. It suggests that, accompanied with the crystallinity increasing, the nanograins coarsen with P replaced by Si, which agrees well with the XRD observation. It is reported that CuP clusters have the ability of reducing the atomic migration and pining down the growth of α-Fe^35^. Therefore, by replacing P with Si in the Fe_81_Si_*x*_B_10_P_8−*x*_Cu_1_ alloys, except the inhibiting effect of P on grain growth diminishing, the density of CuP clusters may also decrease which is expected to cause the increase of grain size.

### Structural defects and nucleation sites

**Figure 6:**
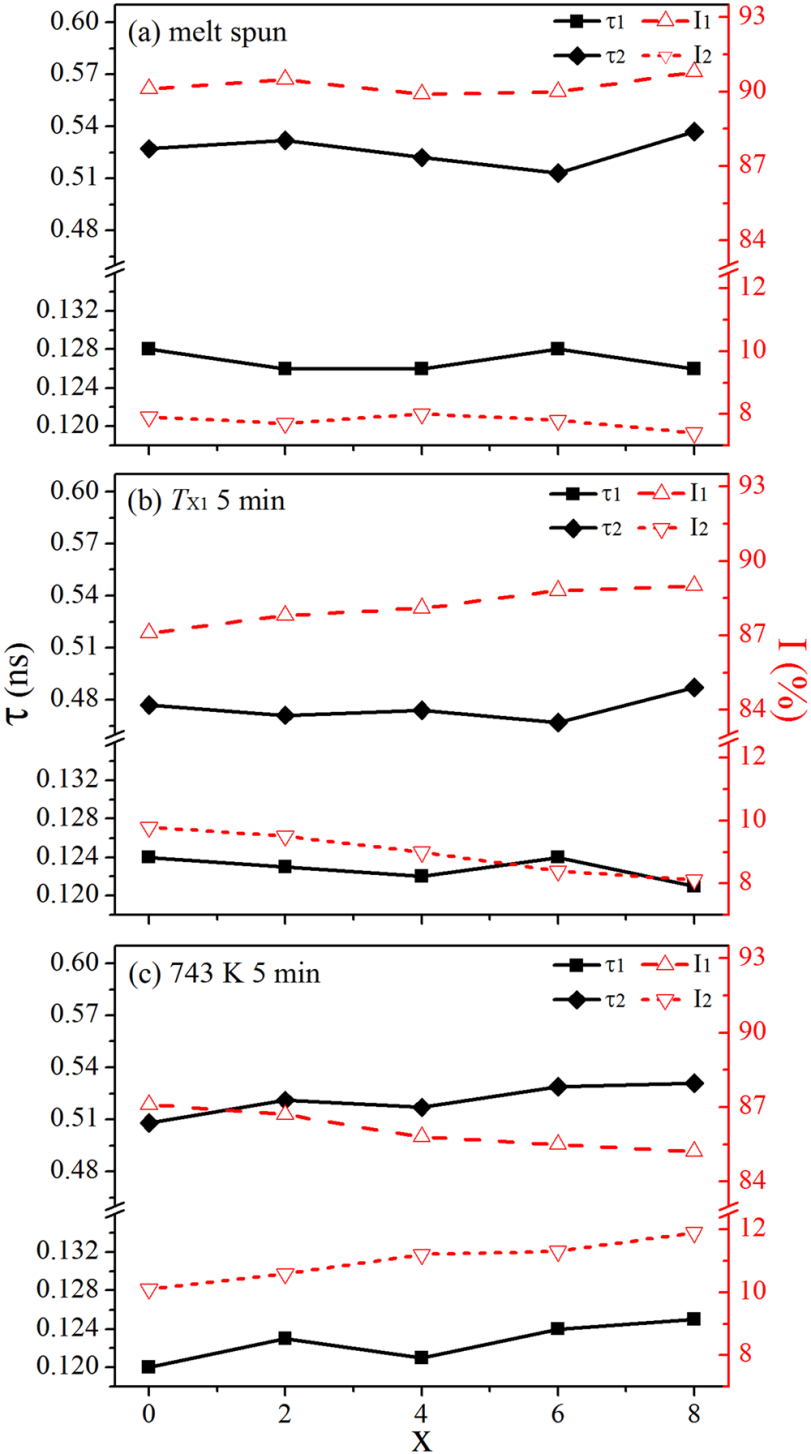
Positron lifetime components *τ*_1_ and *τ*_2_ with their respective intensity *I*_1_ and *I*_2_ of Fe_81_Si_*x*_B_10_P_8−*x*_Cu_1_ (*x* = 0~8) melt spun ribbons (**a**) and those annealed at *T*_X1_ (**b**) and 743 K (**c**) for 5 min.

Structural defects of the Fe_81_Si_*x*_B_10_P_8−*x*_Cu_1_ (*x* = 0, 2, 4, 6 and 8) alloys at various states are analyzed by PALS as shown in Fig. 6. Due to the formation of various free volume defects during the rapid solidification, positron lifetime spectra from all the samples can be best fitted to three lifetime components (*τ*_1_, *τ*_2_ and *τ*_3_) representing three different vacancy defects whose respective intensities are defined as *I*_1_, *I*_2_ and *I*_3_. The relative errors of all the components and their intensities remain in the limit of ±0.5 %. The shortest lifetime *τ*_1_ can be ascribed to the positron lifetime in interstitial holes in the polyhedral packing model which best describes several metallic glasses^36^. The intermediate lifetime *τ*_2_ is related to the positron annihilation in nanovoids in the amorphous matrix and at grain boundaries formed during the annealing^37^. The longest lifetime *τ*_3_ with values of more than 3 ns and intensities of less than 3 % is associated with the ortho-positronium annihilation occurring at the surface between the source and samples, as well as the internal surfaces between the ribbon sheets in the sandwiched samples^38^, and thus it is not given here.

As shown in Fig. 6(a), for the melt spun alloy ribbons, *τ*_1_ values of about 0.127 ns are shorter than 0.175 ns in the pure iron containing crystal mono-vacancy defects^20^, while *τ*_2_ values ranging from 0.513 ns to 0.537 ns indicate the free volume in nanovoids equivalent to at least five atom vacancy clusters in the amorphous structures^39^. A. P. Srivastava *et al*.^40^ and L. Z. Lu *et al*.^41^ have investigated the respective glass-forming ability of Co_69_Fe_*x*_Si_21−*x*_B_10_ and Fe_48−*x*_Co_*x*_Cr_15_Mo_14_C_15_B_6_Y_2_ amorphous alloys by comparing the conspicuous change in sizes of free volume and their intensities. However, *τ*_1_, *τ*_2_ and their corresponding intensities in Fig. 6(a) seem to have no significant fluctuation versus *x*, which implies that there exists no distinct difference in the glass-forming ability of the Fe_81_Si_*x*_B_10_P_8−*x*_Cu_1_ amorphous alloys.

When Fe_81_Si_*x*_B_10_P_8−*x*_Cu_1_ alloys are annealed at their primary crystallization temperature (*T*_X1_) for 5 min, *τ*_1_ and *τ*_2_ values in Fig. 6(b) decrease slightly compared with those in Fig. 6(a), which means the defect sizes in the amorphous matrix reduce due to the structure compaction during the annealing. *I*_2_ associated with the content of nanovoids increases drastically after the annealing, which can be attributed to the formation of numerous crystallites. Therefore, the obvious downward trend of *I*_2_ in Fig. 6(b) illustrates that the content of crystallites at the beginning of crystallization declines with P gradual replaced by Si. It is reported that CuP clusters (Cu_3_P like) separate from the amorphous matrix during the annealing and serve as the heterogeneous nucleation sites for crystallites in FeSiBPCu amorphous alloys^42^. More crystallites will precipitate out with more nucleation sites at the very beginning of crystallization, thus the trend of *I*_2_ in Fig. 6(b) reflects the decrease in the density of heterogeneous nucleation sites in Fe_81_Si_*x*_B_10_P_8−*x*_Cu_1_ alloys, which can be attributed to the reduction of beneficial CuP clusters in the amorphous matrix due to the amount of P decreasing.

Figure 6(c) shows *τ*_1_, *τ*_2_ and their corresponding intensities of the Fe_81_Si_*x*_B_10_P_8−*x*_Cu_1_ nanocrystalline alloys. The value of *τ*_1_ still has no obvious change after the crystallization, while *τ*_2_ increases visually because of the grain growth. The upward trend of *I*_2_ in Fig. 6(c) is ascribed to the increase of grain boundaries versus *x*, which suggests that the crystallinity increases with P replaced by Si in the Fe_81_Si_*x*_B_10_P_8−*x*_Cu_1_ nanocrystalline alloys. It agrees well with the results of XRD and Mössbauer spectroscopy.

### Magnetic properties

**Figure 7:**
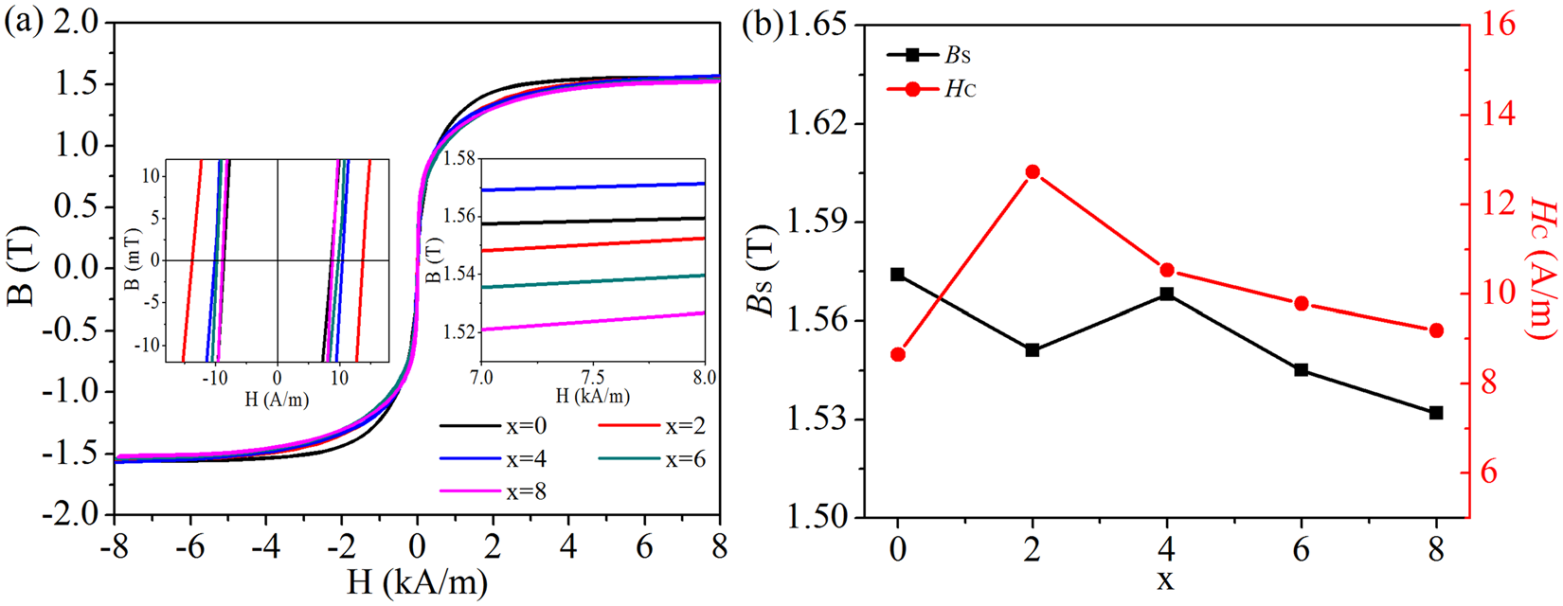
*B*-*H* curves of melt spun Fe_81_Si_*x*_B_10_P_8−*x*_Cu_1_ (*x* = 0~8) ribbons with partially enlarged drawings inserted (**a**) and corresponding variation in *B*_S_ and *H*_C_ versus *x* (**b**).

Figure 7 shows *B*-*H* loops and Si content dependence of *B*_S_ and *H*_C_ of the Fe_81_Si_*x*_B_10_P_8−*x*_Cu_1_ (*x* = 0, 2, 4, 6 and 8) melt spun alloys. It can be found that both Fe_81_B_10_P_8_Cu_1_ and Fe_81_Si_4_B_10_P_4_Cu_1_ melt spun alloys exhibit relatively high *B*_S_, which is consistent with the result of *B*_hf,a_. On the other hand, *H*_C_ initially increases to a maximum in the Fe_81_Si_2_B_10_P_6_Cu_1_ alloy and subsequently decreases, which can be attributed to the variation in magnetic anisotropy associated with the homogeneity of the amorphous matrix observed by Mössbauer spectra.

**Figure 8:**
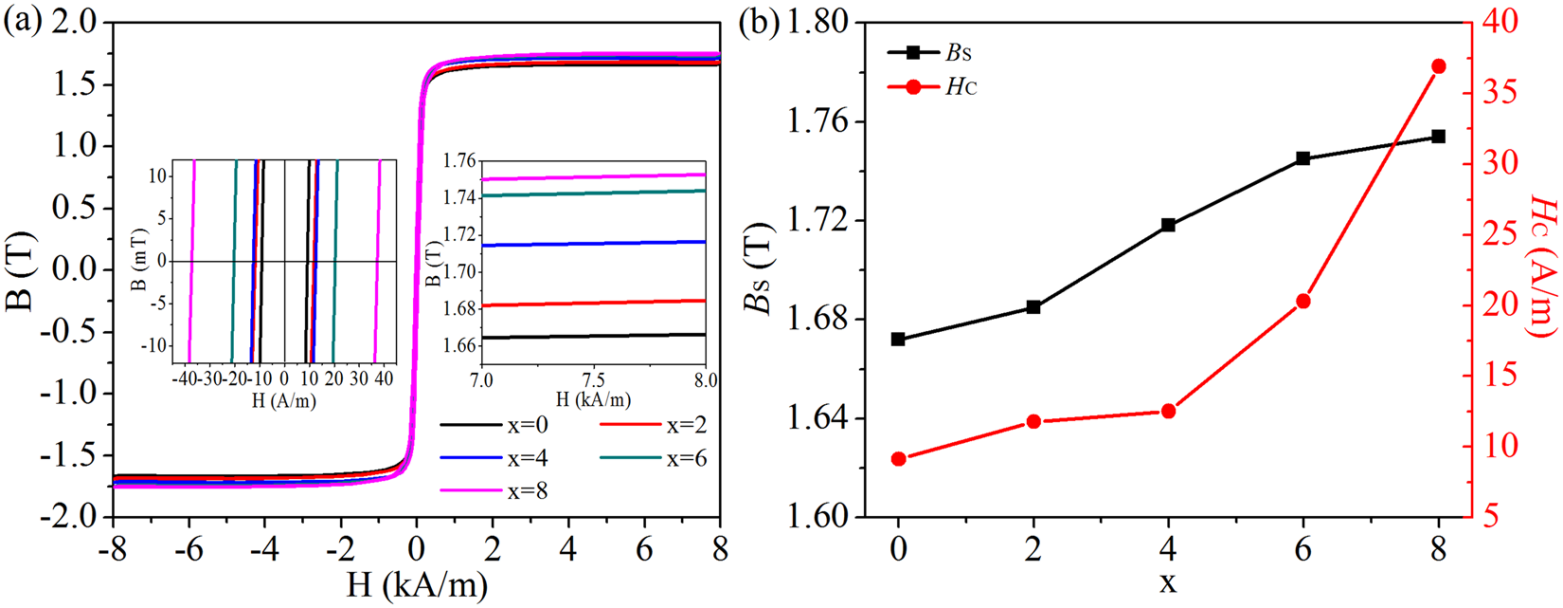
*B*-*H* curves of nanocrystalline Fe_81_Si_*x*_B_10_P_8−*x*_Cu_1_ (*x* = 0~8) ribbons with partially enlarged drawings inserted (**a**) and corresponding change in *B*_S_ and *H*_C_ versus *x* (**b**).

Figure 8 shows *B*-*H* loops as well as *B*_S_ and *H*_C_ versus Si content of the Fe_81_Si_*x*_B_10_P_8−*x*_Cu_1_ (*x* = 0, 2, 4, 6 and 8) nanocrystalline alloys. The value of *B*_S_ of nanocrystalline alloys can be calculated with the following formula^43^:

$${B}_{S}={B}_{SC}R+{B}_{SA}(1-R)$$

where *R* is the crystallinity, *B*_SC_ and *B*_SA_ are the saturation magnetic flux densities of the crystalline and amorphous phases, respectively. Therefore, *B*_S_ strongly depends on the crystallinity *R* because *B*_SC_ is larger than *B*_SA_. On Si substituting P, due to the replacement of Fe by nonmagnetic Si in *bcc* structure, the average magnetic moment of nanocrystallites inevitably decreases. However, the increase of crystallinity dominates with Si substituting for P in the Fe_81_Si_*x*_B_10_P_8−*x*_Cu_1_ alloys, which results in the improvement of *B*_S_ from 1.67 T to 1.75 T. As *H*_C_ ∝ *D*^6^ (*D* is the average grain size)^44^, the increase of *H*_C_ from 9.13 A/m to 36.92 A/m with *x* increasing can be attributed to the grain growth due to the inhibiting effect of P and CuP clusters on grain growth diminishing.

The Fe_81_Si_4_B_10_P_4_Cu_1_ alloy, whether melt spun or nanocrystallized, exhibits simultaneously high *B*_S_ and low *H*_C_ compared with the others. It implies that the appropriate content ratio of Si and P in the Fe_81_Si_*x*_B_10_P_8−*x*_Cu_1_ alloys can not only increase topological structure and homogenize the amorphous matrix, but also provide adequate heterogeneous nucleation sites and promote the crystallinity, all of which contribute to the enhancement of magnetic properties.

## Conclusions

The microstructure of Fe_81_Si_*x*_B_10_P_8−*x*_Cu_1_ (*x* = 0, 2, 4, 6 and 8) alloys and their crystallization mechanism are investigated by Mössbauer spectroscopy and positron annihilation technique. The topological structure and chemical homogeneity vary a lot even if the Fe content remains the same. Meanwhile, the reduction of crystallites at the early stage of crystallization suggests that the density of heterogeneous nucleation sites decreases with the increase of *x* during the annealing, which can reflect the variation in the content of beneficial CuP clusters in the amorphous matrix. Although the grains gradually coarsen with Si substituting for P, the inhibiting effect of CuP clusters and P atoms on the grain growth diminishes leading to the increase of crystallinity. Furthermore, Si tends to occupy lattice positions in *bcc* Fe, which results in the average magnetic moment of nanograins decreasing, but its contribution to *B*_S_ seems to be far less than that from the increase of the crystallinity. Tuning the content of Si and P appropriately in the FeSiBPCu alloys can not only improve the topological structure to strengthen the magnetic interaction in the amorphous matrix and the chemical homogeneity to decrease the magnetic anisotropy, but also optimizes effective CuP clusters during the crystallization to refine nanograins and finally promotes the crystallinity, which is an efficient method to improve synthetical magnetic properties without sacrificing the glass-forming ability.

## Methods

The Fe_81_Si_*x*_B_10_P_8−*x*_Cu_1_ (*x* = 0, 2, 4, 6 and 8) alloy ingots were prepared by induction melting mixtures of industrial raw materials Fe (99.90%), Cu (99.99%), Si-Fe (Si: 99.59%, Fe: 0.27%), P-Fe (P: 26.11%, Fe: 73.80%), B-Fe (B: 17%, Fe: 82.90%) in a Ti-deoxidant argon atmosphere. To ensure the homogeneity of chemical components, they were remelted four times with electromagnetic mixing. Amorphous ribbons with a cross section of about 0.02 × 2.5 mm^2^ were then produced by a melt-spinning technique on a single-roller copper-wheel under argon atmosphere with a surface velocity of about 40 m/s. Thermal properties of melt-spun ribbons were measured by DSC at a heating rate of 20 K/min under high purity argon flow. To investigate the density of heterogeneous nucleation sites at the beginning of primary crystallization, the Fe_81_Si_*x*_B_10_P_8−*x*_Cu_1_ (*x* = 0, 2, 4, 6 and 8) amorphous ribbons sandwiched by two quartz plates were sealed in a high vacuum furnace and isothermally annealed at their individual primary crystallization temperature *T*_x1_ obtained from DSC curves for 5 min by furnace cooling. Nanocrystalline ribbons were obtained by annealing the melt spun alloy ribbons at 743 K for 5 min. All the annealing is carried out with a heating rate of 1 K/s.

The microstructure of melt spun and annealed ribbons was characterized using both XRD with Cu Kα radiation (λ = 0.154056 nm) and room-temperature Mössbauer spectroscopy with ^57^Co as γ-ray source. Velocity calibration of Mössbauer spectroscopy was accomplished by an α-Fe foil of 25 μm thickness. With assuming a distribution of hyperfine fields, Mössbauer spectra were analyzed by a least-square fitting procedure with NORMOS program^45^. TEM specimens were prepared adopting ion thinning at both sides of ribbons and then investigated by Tecnai 12 at 120 kV. PALS was employed to obtain detailed information about the free volume in melt spun and annealed ribbons. Specimens for PALS measurement were composed of two stacks of 10 layers of the sample ribbon with the ^22^Na source sandwiched between them to ensure the annihilation of positrons within the volume of samples. Positron annihilation lifetime spectra had 10^6^ counts and the time resolution of the spectrometer was 230 ps. Each positron lifetime was fitted in three components by using LT software version 9^46^. *H*_C_ and *B*_S_ of samples were measured by DC *B*-*H* loop tracer.
